# Autism spectrum disorder knowledge scale: Chinese revision of the general population version

**DOI:** 10.1186/s12888-023-04538-w

**Published:** 2023-01-25

**Authors:** Linfei Su, Zehui Lin, Youyuan Li, Ling Wei

**Affiliations:** Department of Applied Psychology, School of Health, Fujian Medical University, Fuzhou, China

**Keywords:** Autism spectrum disorder, Knowledge, Knowledge scale, General population, Chinese

## Abstract

The general population of China has misconceptions about Autism Spectrum Disorder (ASD). The measurement of ASD knowledge is conducive to conducting widespread scientific publicity. However, China lacks a structurally complete ASD knowledge scale with good reliability and validity. Therefore, this study aimed to introduce a suitable Chinese ASD knowledge scale. Based on 317 participants, this study revised the Chinese version of the Autism Spectrum Disorder Knowledge Scale(ASKSG), assessed its reliability, validity, and psychometric properties, and analyzed the ASD knowledge of the Chinese general population of this subject sample. The results provided support for the Chinese version of the ASKSG as a suitable measure for assessing ASD knowledge and indicated that ASD knowledge in this study’s sample was relatively poor, particularly with regard to etiology and epidemiology.

## Introduction

Autism Spectrum Disorder (ASD) is a neurodevelopmental disorder with the core symptoms of social communication and interaction defects and limited behavior, interest, and activity patterns [1]. The prevalence of ASD is high in both America and China. The 2021 Centers for Disease Control and Prevention (CDC) report indicated that one out of every 44 eight-year-old children (2.3%) was diagnosed with ASD [2]. Although representative large-scale epidemiological research on ASD in China is lacking, the latest report indicated that the prevalence of ASD among children aged six to 12 in China was approximately 0.7% in 2019, encompassing an estimated 700,000 children [3].

These prevalence rates indicate that many children and families in China are affected by ASD. Promoting scientific knowledge of ASD and the affected population among the general public can reduce misunderstanding and stigmatization of ASD, increase opportunities for intervention, and improve self-esteem, quality of life, and social inclusion among children with ASD. Therefore, this study aimed to introduce a comprehensive ASD knowledge scale to assess the general public’s knowledge of ASD in China, understand the deficiencies, and promote widespread scientific publicity. Disease knowledge refers to the personal understanding of the disease. These perceptions are related to the usual understanding of various aspects of the disease, including etiology, exacerbation factors, symptoms, treatment, and prognosis [4]. Similarly, ASD knowledge, also known as beliefs about ASD, ASD awareness, and ASD understanding, refers to the individual’s understanding and knowledge of ASD.

Publicity regarding ASD knowledge in China is not sufficiently comprehensive and structured. As early as 2009 [5], researchers in China realized the importance of measuring ASD knowledge. From 2009 to date, research on ASD knowledge in China has gradually developed. A scale is one way to measure ASD knowledge. Chinese researchers have developed several scales, including the Mental Health Questionnaire for Children Aged 2–6 Years Old developed by Wang et al. in 2009 [5], the Autism-Related Information Awareness Questionnaire [6], the Autism-Related Information Awareness Survey edited by Zhao et al. in 2017 [7], the Autism-Related Information Awareness Questionnaire for Children edited by Zhang et al. in 2019 [8], and the Chinese version of the Autism Stigma and Knowledge Questionnaire (ASK-Q) edited by Yu et al. [9]. In terms of disease classification, etiology, symptoms, diagnosis, prognosis, and understanding channels, these studies found that most people in China think that ASD, which is actually a neurodevelopmental disorder, is a mental disease [6, 8]. The public consider ASD being mainly due to improper education [6], but the cause of ASD is not currently known [10]. The public considers ASD is wordless [7], but the core symptoms of ASD are social communication disorder and restricted, repetitive behaviors and interests [1]. Fortunately, attitudes toward diagnosis and treatment are positive, and the public thinks that people should consult or seek medical treatment immediately if they find that their child has related symptoms [7, 8], which is accurate. However, the public thinks that the Department of Psychology deal with ASD [6], whereas it is actually the Department of Rehabilitation or the Department of Growth and Development. In addition, the public believes that early treatment and intervention of ASD can improve the condition among children [8], which is correct [11]. The main channels to obtain understanding are newspapers, magazines, and books [7]. These findings indicate that Chinese people retain many misunderstandings regarding ASD. In 2016, a scale with robust psychometric properties was proposed. The operational definition of the criteria were that the reliability and validity of the measurement should be carefully tested. The detection results of the reliability and validity were good [12]. However, most of the above-mentioned scales, except for the ASK-Q, did not demonstrate good psychometric properties. ASD knowledge is vital to the general population [13]. As such, it is crucial to measure the ASD knowledge of the general population, which can be done with the Chinese version of the ASK-Q. However, the Chinese version of ASK-Q is not sufficiently comprehensive to introduce relevant information about the disease. Therefore, this study aimed to screen and revise other internationally established scales.

Internationally available ASD knowledge scales for the general population with robust psychometric properties include Beliefs About Autism developed by Furnham and Buck in 2003, the ASK-Q developed by Harrison et al. in 2017 [14], and the Autism Spectrum Knowledge Scale-General (ASKS-G) developed by McClain et al. in 2019 [13]. Beliefs about autism examines the causes and treatments and is inconsistent with the newly released Diagnostic and Statistical Manual DSM-5 [1]. It describes only autism, whereas other subtypes of ASD are not included. The ASK-Q contains four subscales assessing etiology, symptoms/diagnosis, treatment, and stigma identification but does not incorporate recent epidemiological data on the etiology subscale, such as prevalence and male-to-female ratio, and the Treatment subscales describe the factors affecting treatment incompletely (only age is mentioned). However, the Autism Spectrum Disorder Knowledge Scale(ASKSG) is consistent with DSM-5 and covers a broader field of disease contents, including etiology and epidemiology, symptoms and related behaviors, evaluation and diagnosis, treatment, results and prognosis [15], which is in line with the comprehensive and structured introduction to disease knowledge [4]. Based on the comprehensiveness and structural integrity of the ASKSG, we selected the ASKSG for localization revision.

McClain et al. [16] developed two ASD knowledge scales based on different populations: the Autism Spectrum Knowledge Scale Professional Version-Revised (ASKSP-R), which is used to measure the ASD knowledge of professionals, and the ASKSG, which is used to measure the ASD knowledge of the general population. The ASKSG contains 31 items presented as descriptive declarative sentences. The participants respond with the following options: “True”, “False”, or “Don’t know.” Points are given for correct answers, and “Don’t know” is recorded as a wrong response. Higher scores indicate higher levels of ASD knowledge level. The ASKSG meets the psychometric criteria proposed by Harrison et al. [12]. According to item response theory, the ASKSG is unidimensional (all MSQs < 1.5) with acceptable internal consistency (α = 0.73 raw score, α = 0.75 standardized; λ6 = 0.80). It passed the review of three ASD experts in the fields of clinical and school psychology in support of face validity [13]. In addition, the effectiveness of the ASKSG has been tested with the general American population [15] and the an American parent sample [17]. Furthermore, the ASKSG has been used to test the effectiveness of an ASD knowledge video intervention [18], demonstrating its applicability.

### The present study

This study aimed to revise a Chinese version of the ASKSG and to assess its reliability, validity, and psychometric properties. In addition, we determined the level of knowledge of ASD in a sample of the Chinese general population.

## Methods

### Participants

This study included 317 participants (Table 1), and the inclusion criterion was age 18 years of age or older. The gender ratio of the sample population was comparable to the overall population of China. However, on average, the participants were more educated than the general population average. The subjects in this study mainly included students, medical staff, kindergarten teachers (general schools), primary school teachers (general schools), special education teachers, professoriat, and others including farmers, civil servants, company employees, housewives, etc. The demographics of the present sample and the overall population of China were compared using the Seventh National Census of 2020 from the National Bureau of Statistics of China.

**Table 1:** Demographic information of the subjects (*N* = 317)

	*M*(*SD*)
Age	30.8(10.0)year
	*n*(%)
Gender	
Male	163(51.4)
Female	154(48.6)
Marital status	
Unmarried	152(47.9)
Married	162(51.1)
Divorce	3(0.9)
Education	
High school and below	34(10.7)
Universities and colleges	52(16.4)
Undergraduate	159(50.2)
Master	67(21.1)
Doctor	5(1.6)
Have children or not?	
Have	152(47.9)
Not have	165(52.1)
Occupation	
Student	107(33.8)
Medical staff	14(4.4)
Kindergarten teacher (general school)	3(0.9)
Primary school teachers (general schools)	12(3.8)
Special education teacher	15(4.7)
Professoriat	16(5.0)
Others	150(47.3)
Experience of contact with ASD	
Have	147(46.4)
Not have	170(53.6)

### Revision of the ASKSG

To investigate the knowledge of the general population regarding the prevalence of ASD in China, Item 1, “Less than 2% of people in the US have autism spectrum disorder,” was revised to “Less than 2% of people have ASD,” according to the median prevalence of ASD (1/100) [19], as there are no large-scale epidemiological surveys in China. In addition, with consent from the author of the original ASKSG, the answer to Item 12, “Some individuals with autism spectrum disorder may be uncoordinated or clumsy,” was revised from “False” to “True,” as the original answer was incorrect and was revised by McClain et al. in 2021 [20].

The translation method chosen for this study was the classical back-translation method used by Brislin (1976) [21]. The translation process in this study was as follows: the scale was first translated by two psychology master’s students with strong bilingual skills; then two psychologists with high English proficiency performed cultural adaptation (forward-translation, synthesis, and back-translation) to ensure the accuracy of the translation. One of them is not only an expert in psychology, but also has experience working with special education in China and the United States, which makes her could translate the questionnaire better. And all the translators had an experience in translation. We then obtained the first draft of the scale, followed by a pre-experiment: 20 college students who had not been exposed to the original scale were selected for the pre-survey, and were informed of the purpose and significance of the study before filling out the questionnaire. Next, we repeatedly evaluated the results of the pre-survey regarding content, semantics, criteria, and requirements of each item to create the preliminary Chinese version of the ASKSG.

### Data collection and procedures

A random sample was used for the survey, which consisted of a paper version of the questionnaire and an electronic version. Regarding the paper version of the questionnaire, we used a poster to recruit participants and gathered those who were willing to participate in a large, quiet classroom. Researchers were uniformly trained and the purpose of the study was explained to the participants using uniform guidelines. After obtaining the participant’s consent, they completed the questionnaire independently. Once they were finished we collected the questionnaires. For the online survey we used *Wenjuanxing* (https://www.wjx.cn) because some people lived far away from the site where we were collecting data or it was not convenient to meet. Wenjuanxing is a free online questionnaire and assessment platform. Researchers are able to design questionnaires online, independent of the number of questionnaires, time, and location, which is more efficient and convenient, and is widely used in China. All participants answered some questions about social and demographic information and completed the ASKSG. A total of 317 questionnaires were collected in this study, including 185 paper questionnaires and 132 electronic questionnaires. Berinsky et al. [22] suggested using a filter to assess participants’ attention. Therefore, to improve the validity of the data, an attention check item (#17) was included in the questionnaire that stated the following: “Please select the second answer. (A) True, (B) False, (C) Don’t know.“ Data were included in the analysis only if the participant answered the correct item.

### Data analysis

First, we used the Rasch model in item response theory (IRT) to analyze structural validity. Then, we used Cronbach’s α to test the internal consistency of the scale. Furthermore, we used the Rasch model to analyze the difficulty distribution of the items and the knowledge level distribution of the participants. Analyses were conducted using R 4.1.1 [23] and the “erm” package [24]. Finally, descriptive statistics and the independent samples t-test were used to analyze current ASD knowledge.

## Results

### Reliability and validity

For data analysis, the participants’ responses to the items were coded in binary mode (1 for correct responses and 0 for wrong responses). As the response of “Don’t know” indicated that the participants did not know the correct answer, it was scored as 0 [14]. Rasch model under the framework of IRT was used for analysis. According to the mean square fit statistics parameters (mean square error [MSQ], including Infit and Outfit), we found that the Outfit of Item 21 exceeded 1.5; therefore, we deleted Item 21 and re-analyzed the data. The result showed that the ASKSG was one-dimensional (all MSQs < 1.5). The Cronbach’s α coefficient of the 30 items indicated that the scale’s internal consistency reliability was good (α = 0.834).

### Difficulty and knowledge level distribution

The IRT analysis simultaneously estimated each item’s difficulty (β) and knowledge level (θ) of each individual using the maximum likelihood method. With increasing difficulty of the item, only the participants with higher knowledge level (θ) gave the correct response. Table 2 shows the sample response correct rate for each item and the difficulty parameter (*η*) estimated on the continuous logit scale. In addition, the model estimated the ASD knowledge level (θ) required for a participant given a 50% chance of correctly responding to an item.

**Table 2:** ASKSG Items, Sample Percentage of Correct Responses and Estimated Item Difficulty from Rasch Model

NO.	Accuracy (%)	Item difficulty parameter(*η*)	
		Est.	SE
11	92.11	-2.875	0.219
13	85.80	-2.135	0.172
12	81.39	-1.759	0.156
28	78.55	-1.550	0.149
17	76.97	-1.442	0.145
8	76.34	-1.400	0.144
27	76.03	-1.379	0.144
30	74.45	-1.278	0.141
31	74.45	-1.278	0.141
20	71.61	-1.105	0.137
24	71.61	-1.105	0.137
10	71.29	-1.086	0.136
26	70.98	-1.068	0.136
29	69.72	-0.995	0.134
5	66.56	-0.819	0.131
4	57.10	-0.330	0.125
25	54.26	-0.190	0.124
15	53.63	-0.159	0.124
14	53.31	-0.143	0.124
16	50.16	0.010	0.124
19	50.16	0.010	0.124
2	49.84	0.026	0.124
3	49.53	0.041	0.124
9	49.53	0.041	0.124
23	41.96	0.410	0.125
18	37.54	0.631	0.127
7	25.87	1.272	0.138
6	21.14	1.575	0.147
1	20.19	1.641	0.149
22	15.77	1.980	0.163

Items in Table 2 are ranked according to increasing difficulty from top to bottom. The first column number indicates the order in which items appear in the ASKSG

Items in Table 2 are ranked according to increasing difficulty from top to bottom. The first column number indicates the order in which items appear in the ASKSG.

According to the difficulty coefficient, the simplest item was Item 11, “Individuals with ASD have difficulty communicating with others,” and 92% of the participants responded to this item correctly (*η*= -2.875). A moderately difficult topic was Item 16, “Autism spectrum disorder can only be diagnosed after the age of four,” and 50% of the participants responded to this item correctly (*η* = 0.010). The most difficult item was Item 22, “There is no effective treatment for autism spectrum disorder at present,” and 16% of the participants responded to this item correctly (*η* = 1.980).

**Fig. 1:**
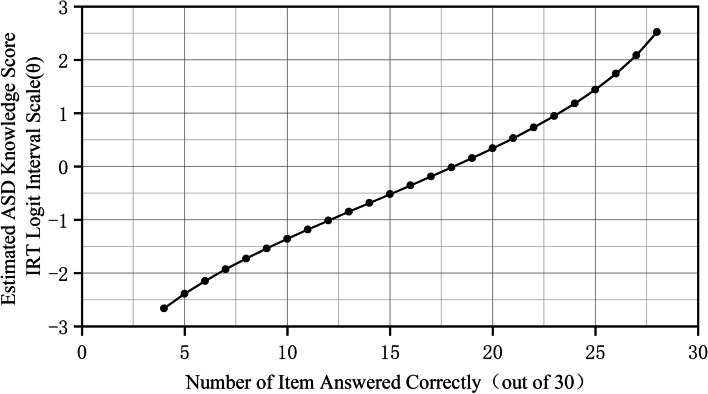
Score interval for ASD knowledge (θ) transformed from raw correct count visualization of 30 items to logit scale

The total number of correct responses was calculated out of 30 items and converted into a θ score on the logit scale (Table 3), which is regarded as a continuous equidistant/proportional scale. This transformation was nonlinear in the visualization presented in Fig. 1, which indicated that using the number or percentage of items correctly answered by the participants (the items are regarded as equal weights) was appropriate as an index to evaluate individual ASD knowledge level.

θ value is based on logit's estimation of accurate scale rather than strict ordinal value.

**Table 3:** On the logit scale of Rasch model, the conversion from the correct items answered to the estimated ASD knowledge level (θ)

Correct quantities of 30	Accuracy of 30 items (%)	Estimated ASD knowledge score(θ)
4	13.33	-2.660
5	16.67	-2.385
6	20.00	-2.144
7	23.33	-1.926
8	26.67	-1.725
9	30.00	-1.535
10	33.33	-1.355
11	36.67	-1.181
12	40.00	-1.012
13	43.33	-0.846
14	46.67	-0.682
15	50.00	-0.517
16	53.33	-0.352
17	56.67	-0.185
18	60.00	-0.014
19	63.33	0.161
20	66.67	0.342
21	70.00	0.533
22	73.33	0.735
23	76.67	0.951
24	80.00	1.186
25	83.33	1.446
26	86.67	1.742
27	90.00	2.090
28	93.33	2.526

θ value is based on logit's estimation of accurate scale rather than strict ordinal value.

**Fig. 2:**
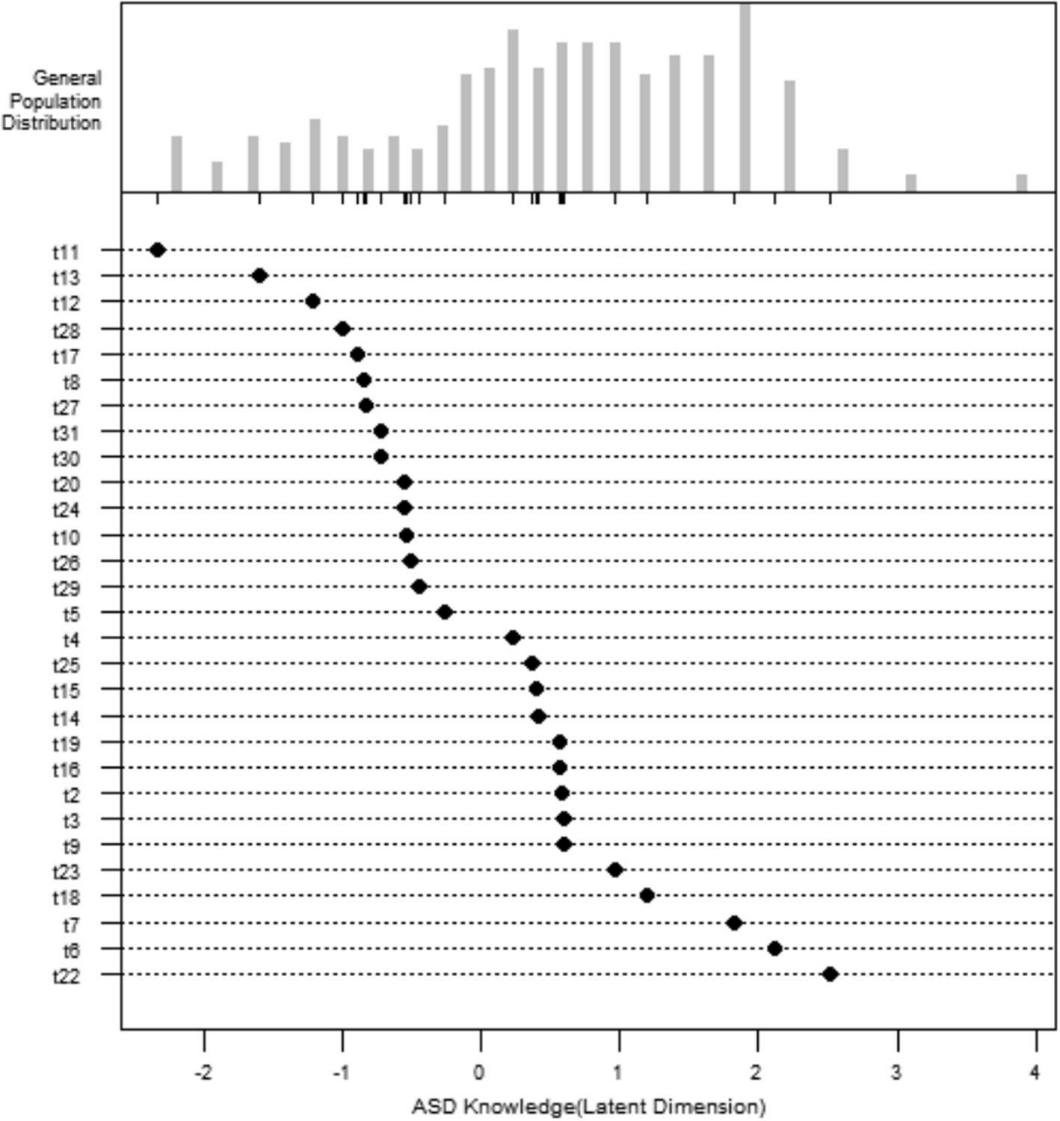
“Person-item diagram” of ASD knowledge

Figure 2 presents the difficulty of all items and distribution of the number of participants at each knowledge level in the same person-item diagram. The scales on the horizontal axis of the upper and lower parts are the same, both on the logarithmic scale. The data points on the horizontal axis corresponding to the black dots at the bottom of the figure represent the difficulty level (*η*) of each item and the ASD knowledge level (θ) required for the participants with a 50% chance of answering the item correctly (the values are the same). The ASKSG items are ranked by difficulty from top to bottom. The histogram at the top of the figure shows the distribution of the number of participants at each knowledge level, with greater knowledge from left to right.

IRT analysis demonstrated that the difficulty of the items (see Table 2; Fig. 1) roughly covered the knowledge range of the people sampled (see Table 3; Fig. 2).

### Status quo of ASD knowledge of the general population in China

Descriptive statistics (including means and standard deviations) were calculated on the correct rate of the whole scale, content areas, and various items. ASD knowledge was analyzed (Tables 4 and 5).The results revealed that the overall correct rate was 58.9%. The correct rate for content areas, from high to low, was as follows: results and prognosis (74.03%), symptoms and related behaviors (72.83%), evaluation and diagnosis (56.68%), treatment (45.90%), and etiology and prevalence (41.46%). No items had a 100% correct rate. The correct rate was over 80% for the following three items: Item 11 (“Individuals with ASD have difficulty communicating with others,” 92.1%), Item 13 (“Many people with ASD have difficulty expressing themselves,” 85.8%), and Item 12 (“Some people with ASD may act incongruously or look clumsy,” 81.4%). The three items with the lowest correct rates were Item 6 (“Having an old father is a risk factor for ASD,” 21.1%), Item 1 (“Less than 2% of people have ASD,” 20.2%), and Item 22 (“There is no effective treatment for ASD at present,” 15.8%). As these three items are the most difficult items,the participants who responded correctly to these three items may have had a higher level of ASD knowledge.

**Table 4:** Descriptive statistics of accuracy rate of content areas

Content area	*M*(%)	*SD*(%)
Full scale	58.9	19.2
Etiology and epidemiology	41.5	22.9
Symptoms and related behaviors	72.8	23.2
Evaluation and diagnosis	56.7	27.6
Treat	45.9	26.4
Results and prognosis	74.0	25.9

In descending order of correct rate

**Table 5:** Descriptive statistics of the accuracy rate of the items

NO.	*M*(%)	*SD*(%)
11	92.1	27.0
13	85.8	35.0
12	81.4	39.0
28	78.5	41.1
17	77.0	42.2
8	76.3	42.6
27	76.0	42.8
30	74.4	43.7
31	74.4	43.7
20	71.6	45.2
24	71.6	45.2
10	71.3	45.3
26	71.0	45.5
29	69.7	46.0
5	66.6	47.3
4	57.1	49.6
25	54.3	49.9
15	53.6	49.9
14	53.3	50.0
16	50.2	50.1
19	50.2	50.1
2	49.8	50.1
3	49.5	50.1
9	49.5	50.1
23	42.0	49.4
18	37.5	48.5
7	25.9	43.9
6	21.1	40.9
1	20.2	40.2
22	15.8	36.5

Sort by correct rate

Gender, marital status, presence of children, and experience with ASD individuals were used as grouping variables. The accuracy rate of the whole scale was used as the analysis index for the independent sample t-test (Table 6). The results showed that the correct rate for the full scale among women was significantly higher than among men (63.79 ± 17.98 and 54.34 ± 19.15, respectively; *t* = -4.525, *p* < 0.001). Married participants had a significantly higher accuracy on the full scale than unmarried people (65.21 ± 17.93 and 52.24 ± 18.25, respectively; *t* = -6.350, *p* < 0.001). Participants with children had a significantly higher rate on the full scale than those without children (65.96 ± 17.14 and 52.44 ± 18.66, respectively; *t* = 6.701, *p* < 0.001). Participants with ASD experience had a significantly higher rate on the full scale than those without ASD experience (68.71 ± 15.32 and 50.47 ± 18.11, respectively; *t* = 9.711, *p* < 0.001).

**Table 6:** The accuracy of the full scale of subjects with different gender, marital status, whether they have children or not, and experience of contact with ASD

Grouping variables	Group	*N*	*M*(%)	*SD*(%)	*t*	*p*
Gender	Male	163	54.34	19.15	-4.525	0.000
	Female	154	63.79	17.98		
Marital status^*^	Married	162	65.21	17.93	-6.350	0.000
	Unmarried	152	52.24	18.25		
Have children or not	Have	152	65.96	17.14	6.701	0.000
	Not have	165	52.44	18.66		
Experience of contact with ASD	Have	147	68.71	15.32	9.711	0.000
	Not have	170	50.47	18.11		

^*^ In the marital status, only the differences between married and unmarried groups are analyzed. The divorced and widowed groups are not included in the analysis because of their small number (3 people in total)

## Discussion

After preliminary development and analysis, the internal consistency reliability coefficient of the ASKSG was good, indicating that the ASKSG may be a reliable measure of ASD knowledge in the general population in China. Regarding validity, the results of the IRT analysis provided acceptable evidence that the measurement was sufficiently one-dimensional and consistent with the original scale, further suggesting that the ASKSG may be a reliable and effective method to measure ASD knowledge among Chinese population. In addition, the IRT analysis revealed that the difficulty of the items roughly covered the knowledge range of the sampled population, indicating that the measure could appropriately identify the level of ASD knowledge of individuals.

The correct rate in this study was 58.9%, with three items with a correct rate of over 80%, whereas the general population in the United States had a correct rate of 64.3%, with eight items with a correct rate of over 80% [15]. Moreover, neither the present study nor the study in United States had a correct rate of 100%. Such rates suggest that these populations have a relatively limited knowledge of ASD. The accuracy order in the present study by content, from high to low, was results and prognosis, symptoms and related behaviors, evaluation and diagnosis, treatment, then etiology and epidemiology. In the United States, the highest accuracy was for symptoms and related behaviors and the lowest was for evaluation and diagnosis [15]. This indicates differences in the knowledge structure between the two populations. The levels of knowledge about ASD in the present study and in the United States were similar and relatively insufficient. Moreover, the knowledge structure in these populations was different. The present study revealed greater knowledge regarding outcome and prognosis and less knowledge regarding cause and prevalence. Therefore, future public ASD education in China should focus on cause and prevalence.

In addition, this study revealed that knowledge of ASD was significantly higher among women than men. This is consistent with a report that showed Chinese women had a higher demand for mental health knowledge in educating children and preventing and treating mental diseases and a more active and urgent need for mental health knowledge than men [25]. China should strengthen men’s attention to and promotion of ASD knowledge. ASD knowledge among married participants was significantly higher than those who were unmarried. Due to pregnancy preparation, married people may be exposed to more ASD-related knowledge, such as the relationship between food intolerance and ASD [26]. ASD knowledge of participants with children was significantly higher than that of participants without children. This finding is consistent with a survey that demonstrated children’s mental health was the second biggest concern of parents (their primary concern was physical health). The knowledge level of participants with experience with ASD individuals was significantly higher than that of participants without experience with ASD individuals. According to the exposure effect or the mere exposure effect [27], exposure to individuals who have ASD individuals may make people more willing to accept them and take the initiative to learn about ASD. As places outside of the homes of individuals with ASD are relatively limited, such as special education schools or rehabilitation institutions (according to the White Paper on the Investigation of Autistic Families in China in 2021). To address this lack of exposure, enhanced forms of publicity could include making animations to better reflect the real life of individuals with ASD, increasing indirect contact with the general population, and encouraging the general population to pay attention to and understand ASD.

### Limitations and future research

The first limitation is the representativeness of the participants. This study included participants from a variety of occupations, including college students, teachers, parents with ASD, farmers, civil servants, and housewives. This relatively diverse sample can represent the knowledge level of some of the general public; however, most of the participants had a high level of knowledge, so there are limitations with regard to generalizing the findings to Chinese citizens with a lower level of education.

The second limitation of this study is the translation of the scales. The translation method chosen for this study was the classical back-translation method used by Brislin (1976) [21],  As mentioned by Vujcich et al. (2021) [28], forward-backward translation represents an attempt to overcome the risks inherent in relying on a single individual. However, a criticism of forward-backward translation is that it has the potential to focus too narrowly on the task of literal translation at the expense of ensuring that the translation captures the intended meaning of the survey item in a way that is clear and suitable for the intended audience [29]. Therefore other better translation methods can be tried in the future, such as the TRAPD used in the study of Vujcich et al. (2021) [28]. The Best Practice Guidelines for Cross-Cultural Surveys recommends “team translation,“ particularly the approach known as TRAPD, the version endorsed in the Best Practice Guidelines for Cross-Cultural Surveys, which is considered to be the better translation approach.This is because “team translation” allows people with complementary knowledge and expertise to work together to achieve the best possible translation to ensure that survey items convey what they are intended to convey to the target audience [30, 31].

A final limitation was the analysis regarding the reliability and validity of the scale. In terms of reliability, this study considered evaluating test-retest of reliability;however, because many participants were unable to be contacted a second time for retesting, we thought conducting test-retest reliability would be problematic in this study. In terms of validity, given the response options on the scale are binary, and that it has been mentioned in the literature that binary items are not well suited for exploratory factor analysis [32], we used a more appropriate analysis method to evaluate validity, which is IRT. IRT was the same analysis method used in the original scale.

In future research, the ASKSG can be used as a measure of the effectiveness of ASD knowledge intervention methods, such as PowerPoint, video, and on-site teaching, and the relationship between explicit changes (i.e., knowledge) and implicit changes (e.g., attitude) can be analyzed by combining explicit and implicit measures. Future studies could also focus on ASD knowledge promotion according to the general population’s understanding of different aspects of knowledge assessed by the ASKSG.

## Conclusion

This study revised the Chinese version of the ASKSG, which is the first autism spectrum disorder knowledge scale in China. It demonstrated good reliability and validity, and can be considered as an evaluation indicator to measure the level of knowledge about autism spectrum disorders among the general public in China, in order to study the knowledge of the general public about autism-related disorders in China. It is hoped that the present study can contribute to improving the understanding, awareness, and tolerance of autism among the general public and draw attention to and benefit people with ASD.

## Data Availability

The datasets generated and/or analysed during the current study are not publicly available due to some special reasons but are available from the corresponding author on reasonable request.
